# Selective mitochondrial DNA degradation following double-strand breaks

**DOI:** 10.1371/journal.pone.0176795

**Published:** 2017-04-28

**Authors:** Amandine Moretton, Frédéric Morel, Bertil Macao, Philippe Lachaume, Layal Ishak, Mathilde Lefebvre, Isabelle Garreau-Balandier, Patrick Vernet, Maria Falkenberg, Géraldine Farge

**Affiliations:** 1 Université Clermont Auvergne, CNRS/IN2P3, Laboratoire de Physique de Clermont, BP 10448, F-63000 Clermont-Ferrand, France; 2 Institute of Biomedicine, University of Gothenburg, P.O. Box 440, SE-405 30, Gothenburg, Sweden; Instituto de Biologia Molecular de Barcelona, SPAIN

## Abstract

Mitochondrial DNA (mtDNA) can undergo double-strand breaks (DSBs), caused by defective replication, or by various endogenous or exogenous sources, such as reactive oxygen species, chemotherapeutic agents or ionizing radiations. MtDNA encodes for proteins involved in ATP production, and maintenance of genome integrity following DSBs is thus of crucial importance. However, the mechanisms involved in mtDNA maintenance after DSBs remain unknown. In this study, we investigated the consequences of the production of mtDNA DSBs using a human inducible cell system expressing the restriction enzyme PstI targeted to mitochondria. Using this system, we could not find any support for DSB repair of mtDNA. Instead we observed a loss of the damaged mtDNA molecules and a severe decrease in mtDNA content. We demonstrate that none of the known mitochondrial nucleases are involved in the mtDNA degradation and that the DNA loss is not due to autophagy, mitophagy or apoptosis. Our study suggests that a still uncharacterized pathway for the targeted degradation of damaged mtDNA in a mitophagy/autophagy-independent manner is present in mitochondria, and might provide the main mechanism used by the cells to deal with DSBs.

## Introduction

Mitochondria are cellular organelles that possess their own circular genome. The mitochondrial DNA (mtDNA) is compact, with no introns, and contains only 37 genes. Therefore, most of the proteins that are acting in mitochondria are encoded in the nucleus and imported into the mitochondria. Point mutations, deletions, but also depletion of mtDNA, are found in numerous pathologies, such as mitochondrial diseases [1], cancers [2], neurodegenerative disorders [3] and even during the normal ageing process [2, 4]. As oxidative phosphorylation takes place in the vicinity of the mitochondrial genome, mtDNA is believed to be prone to oxidative damage due to reactive oxygen species (ROS) [5]. Other mtDNA lesions, such as DNA breaks, can also occur during replication or repair of mtDNA [6]. If DNA damage is left unrepaired or is not correctly repaired it can lead to mutations, therefore accurate mtDNA maintenance systems are essential for cell viability. In mitochondria, damage such as oxidative damage is mostly repaired by Base Excision Repair (BER). The proteins involved are well characterized and are mostly the same proteins as in the nuclear BER [7]. However, it remains unclear which mechanisms are responsible for mtDNA maintenance after double-strand breaks (DSBs). In *Saccharomyces cerevisiae* mtDSBs are repaired by homologous recombination, with the involvement of members of the RAD52 epistasis group [8]. Evidence of mtDSB repair has also been found in *Drosophila* cells [9]. However, although it has been shown that some of the factors needed for DSB repair in the nucleus are present in mammalian mitochondria, for example XRCC1 [10] or an alternate form of Ku80 [11], it is still debated whether or not mtDSBs are repaired in mammalian cells, and, if so, by which mechanisms. Some studies suggest that recombination occurs after mtDSBs, with intramolecular recombination being more frequent than intermolecular recombination [12]. In mouse models, it has been suggested that mtDSBs are repaired by non-homologous end-joining (NHEJ), with a region close to the D-loop being involved in most recombination events [13]. In contrast, another study has proposed that classical NHEJ is undetectable, but that instead microhomology-mediated end-joining (MMEJ) has a central role in the maintenance of mtDNA integrity after DSBs [14]. This result is supported by a study showing that around 85% of deletions in mtDNA of human cells are flanked by short repetitive sequences, suggesting the existence of recombination events [15].

A different scenario is that, after the generation of mtDSBs, the damaged mtDNA molecules undergo degradation. In most cells there are indeed thousands of mtDNA molecules, and the elimination of some of them should not compromise mitochondrial function. Little is known about the mechanisms of degradation of mtDNA which seems to occur in response to damage that mitochondria cannot repair [16, 17]. Different processes with different kinetics have been suggested, depending on the cell type [18]. The two most likely mechanisms proposed are (i), the degradation of the damaged mtDNA by nucleases and (ii), the elimination of the mitochondria carrying damaged mtDNA by autophagy/mitophagy. The actors of mtDNA degradation are currently unknown. To date, five DNA nucleases have been localized in the mitochondria: ExoG, EndoG, MGME1, DNA2 and FEN1, but their exact functions are still not completely elucidated [19]. ExoG is exclusively mitochondrial and has been implicated in BER and mitochondrial single-strand break repair [20]. EndoG is located in the mitochondrial intermembrane space and under certain conditions relocates to the nucleus where it could play a role in apoptosis [21] and in recombination [22]. MGME1, DNA2 and FEN1 are believed to be involved in mitochondrial primer processing during replication [23]. Moreover, MGME1 could also be implicated in intramolecular recombination of mtDNA [24] and in 7S DNA turn-over [25], whereas DNA2 and FEN1 may also play a role in mitochondrial BER [26, 27]. Damage repair or degradation of mutated mtDNA are not the only possibilities for the organism or the cell to maintain the integrity of its genome. If damage cannot be repaired, signaling pathways can also trigger apoptosis, leading to cell death, or induce fission mechanisms to segregate mitochondria with overwhelming damage, which will be eliminated by autophagy/mitophagy [28, 29].

There are three main techniques that have been developed to manipulate and create DSBs specifically in the mitochondrial genome [30]. In the first technique, restriction enzymes preceded by a mitochondrial targeting sequence (MTS) have been used to cut mtDNA at specific restriction sites [31]. Following this strategy, the use of the endonuclease EcoRI in human, mouse and rat cell lines has led to the generation of *ρ*^0^ cells, devoid of mtDNA [32]. Mouse cells stably expressing mitochondrial SacI showed a similar pattern, with depletion of mtDNA but without a total loss. This strategy was also used *in vivo* on a mouse model containing two polymorphic mtDNA sequence variants, resulting in either three or five restriction sites for SacI. After injection of recombinant viruses encoding for mitochondrial SacI in these animals, intra- and inter-molecular recombination events following mtDSBs were observed [13]. The two other techniques are based on zinc-finger nucleases (ZFNs) and transcription activator-like effector nucleases (TALENs). These two nucleases contain two domains, a DNA-cleaving domain and a DNA-binding domain specific to the targeted DNA sequence. All these techniques have mainly been used to develop therapeutic tools to cure mitochondrial diseases. Within the same mitochondria, mutated DNA can coexist with wild-type DNA, a phenomenon called heteroplasmy. With mtDNA disease mutations, the symptoms typically appear only when the heteroplasmy level in the patient’s mitochondria reaches a certain threshold [33]. The preferential targeting of nucleases to mutant mtDNA molecules would thus eliminate them and decrease the heteroplasmy level, providing a possible treatment strategy. The remaining wild-type DNA could then be restored to normal levels through replication [34]. Such a shift in heteroplasmy level has for instance successfully been achieved with a ZFN-based approach in a cell model [35].

In this work we have used a human inducible cell system expressing the restriction enzyme PstI targeted to mitochondria. Induction of PstI resulted in an efficient generation of mtDSBs, followed by a fast loss of the damaged DNA molecules. We found that none of the known mitochondrial nucleases are involved in the mtDNA degradation process and that the DNA loss is not due to autophagy, mitophagy or apoptosis. We further established that the DNA-loss phase is followed by a new amplification of intact mtDNA molecules but found no support for the existence of a DSB repair mechanism in mitochondria.

## Materials and methods

### Transfection and cell culture

The pcDNA3 vector containing the PstI gene flanked by the cytochrome c oxidase subunit VIII mitochondrial targeting sequence was a kind gift of Dr. Carlos Moraes. The construct was re-cloned in pcDNA5/FRT/TO vector (Invitrogen) and the resulting construct was confirmed by DNA sequencing. An inducible stable cell line expressing PstI was created using the Flp-In T-Rex 293 cell line (Invitrogen). Cells were co-transfected with the pcDNA5/FRT/TO construct and pOG44 (Invitrogen) -which encodes the Flp recombinase- at a molar ratio of 1:10 using Lipofectamine 2000 (Invitrogen). Forty-eight hours after transfection Hygromycin (150 μg/mL) (Invivogen) and Blasticidin (15 μg/mL) (Invivogen) were added to the medium for selection. Expression of PstI was induced by adding 1 μg/mL doxycycline to the growth medium for the indicated time.

For siRNA down-regulation experiments, 0.5x10^6^ cells were transfected with a set of 2 or 3 siRNAs (5 to 20nM each) using Lipofectamine RNAiMAX in Opti-MEM medium. After 5h, Opti-MEM medium was replaced by fresh complete medium and cells were used for further analysis 3 days after transfection. The siRNAs used were the following: ExoG: HSS115057 (ThermoFisher Stealth siRNAs), s19298 (Ambion), SI03075534 (Qiagen); EndoG: s707 (Ambion), SI03083878 (Qiagen); MGME1: SI04336976 (Qiagen), HSS132389 and HSS132390 (ThermoFisher Stealth siRNAs); DNA2: HSS141856, HSS141857 and HSS141858 (ThermoFisher Stealth siRNAs); FEN1: HSS103627, HSS103629 and HSS176903 (ThermoFisher Stealth siRNAs). For experiments in which siRNA transfection was followed by PstI induction, doxycycline was added to the cells 3 days after siRNA transfection.

Transgenic cells were grown in Dulbecco’s modified Eagle’s medium (DMEM) with high glucose (GlutaMAX, Gibco), supplemented with 1mM Na pyruvate, 10% FCS (tetracycline free, Biowest), 100U/mL penicillin and streptomycin (HyClone, Thermo Scientific), 150 μg/mL Hygromycin (Invivogen) and 15 μg/mL Blasticidin (Invivogen). The non-transfected Flp-In T-Rex 293 (referred to as wild-type in the experiments) were grown under similar conditions but without Hygromycin.

### Southern blot analysis

Total DNA was isolated from 1-3x10^6^ cells using a NucleoSpin Tissue Kit (Macherey-Nagel). Nucleic acid concentration and purity were measured using a Nanodrop (Nanodrop ND-1000, Thermo Scientific). One microgram of DNA was digested with BamHI or BamHI and PstI. Digestion products were separated on 1% agarose gel and transferred on nylon membrane. The membranes were hybridized with radiolabeled mitochondrial probes and imaged using a phosphoimager (BioRad). Primers sequences used for producing mitochondrial probes were as follows: Hi890 fwd: 5’-CAC GGG CTT ACA TCC TCA TT-3’; Hi890 rev: 5’-TGG CTC AGT GTC AGT TCG AG-3’; Pst fwd: 5’-TTC ATG ATC ACG CCC TCA TA-3’; Pst rev: 5’-TTT TAT GGG CTT TGG TGA GG-3’; Sac fwd: 5’-GCT AA ACCT AGC CCC AAA CC-3’; Sac rev: 5’-AAA CAG GCG GGG TAA GAT TT-3’.

### Real-time quantitative PCR

All reactions were performed on a MasterCycler RealPlex (Eppendorf) using MESA GREEN qPCR Mastermix Plus for SYBR Assay (Eurogentec). Copy numbers of mitochondrial DNA were determined by amplifying by real-time quantitative PCR a portion of the *cytochrome b* gene, using the single copy gene for amyloid precursor protein *APP* as a nuclear standard. The PCR (94°C for 2 min, 40 cycles of 94°C for 20s, 60.4°C for 20s and 72°C for 45s) were performed using 10ng of total DNA and the following primers: cytochrome b fwd: 5’-GCC TGC CTG ATC CTC CAA AT-3’, cytochrome b rev: 5’-AAG GTA GCG GAT GAT TCA GCC-3’; APP fwd 5’-TTT TTG TGT GCT CTC CCA GGT CT-3’, APP rev: 5’-TGG TCA CTG GTT GGT TGG C-3’. Each measurement was performed in duplicate. The specificity of the amplification is controlled by the analysis of the melting curve. The amplification efficiencies were determined with standard curves, performed for both primers pairs with different total DNA dilutions. The ratio of mitochondrial DNA / nuclear DNA was estimated using the cycle thresholds (C*_T_*) values obtained.

The relative level of PstI mRNA was estimated by real-time quantitative PCR using the TBP transcript as an internal control. Total RNA was isolated from 0.5 to 1.5x10^6^ cells using TRI Reagent (Molecular Research Center Inc.) according to the manufacturer’s protocol. cDNAs were synthesized via random hexamer primers (Promega) and M-MLV Reverse Transcriptase (Promega). PCR (94°C for 2 minutes, 40 cycles of 94°C for 20 s, 60°C for 20 s, 72°C for 45 s) were performed using the following primers: PstI fwd: 5’-ATT ACA AGC CCA ATT CCC GC-3’, PstI rev: 5’-TAG GAA CGT GGG TCA GGA AC-3’; TBP fwd: 5’-ACG CCA GCT TCG GAG AGT TC -3’ and TBP rev: 5’-CAA ACC GCT TGG GAT TAT ATT CG-3’. A standard curve for each gene and for the internal control was generated using a dilution series (1:10 to 1:80) of cDNAs consisting of pooled cDNAs from each sample. Each quantification was performed in duplicate.

### Western blot analysis

Total cell lysates were analyzed by SDS PAGE and immunoblotting. Primary antibodies used for western blotting were as follows: ATP5a, UQCR2, SDHB, COII, NDUFB8 (Abcam 110411, 1: 1000); 49KDa (home made, 1: 500); NDUFA9 (Abcam 14713, 1: 1000); H3 (Abcam 10799, 1: 10 000); Twinkle (home made, 1: 1000); TFAM (home made, 1: 5000); exoG (Proteintech 21523-1-AP, 1: 500); MGME1 (Sigma HPA040913, 1: 200); a-tubulin (Sigma T9026, 1: 5000); endoG (Abcam 9647, 1: 1000); Fen1 (home made, 1: 1000); PDH (MitoSciences MSP07, 1: 2500); Tomm20 (Abcam 56783, 1: 500); LC3B (Abcam 48394, 1: 2000); PINK1 (Abcam 75487, 1:1000). Secondary antibodies were as follows: Rabbit (HRP) anti-mouse (Sigma A9044, 1: 10 000) and Goat (HRP) anti-rabbit (Abliance BL2407, 1: 10 000). The detection of proteins was performed using Luminata forte western HRP Substrate (Millipore) or SuperSignal West Femto Maximum Sensitivity Substrate (Thermo Scientific). The relative abundance of specific proteins was evaluated by densitometric quantification of signal intensity, and analyzed using Quantity One image analysis software (BioRad).

### Flow cytometry

All flow cytometry analyses were performed using an Attune Acoustic Focusing Cytometer. For mitochondrial mass measurements, cells were incubated 30 min with 150 nM MitoTracker Green FM (Molecular Probes) and washed with PBS. Cells were then harvested and washed twice with medium without phenol red. 5.10^5^cells/mL were used for each sample. A few minutes before FACS acquisition, cells were incubated on ice in the dark with a vital dye (SYTOX Blue Dead Cell Stain, Life Technologies) to exclude dead cells from the analysis. A negative control was treated with the same procedure but without MitoTracker staining. This negative control represents the background due to cell’s autofluorescence. MitoTracker Green fluorescence was measured using a 488 nm laser and a 530/30 nm filter, and Sytox-blue fluorescence was measured using a 405 nm violet laser and a 450/40 nm filter. On average, 10000 events were analyzed per sample. A two gating strategy was performed to define the target cell population to analyze: first, to exclude dead cells and debris, cells were gated on a two physical parameters dot plot measuring forward scatter (FSC) versus side scatter (SSC), second, Sytox Blue-negative cells are gated. The mitochondrial mass was evaluated by the mean fluorescence intensity of the MitoTracker channel.

Cellular apoptosis was measured using the Dead Cell Apoptosis Kit with Annexin V Alexa Fluor 488 & Propidium Iodide (PI) (Molecular Probes) according to manufacturer’s instructions. A positive control for apoptosis was treated with 2 μM staurosporine for 4h before harvesting the cells. Cells floating in the medium were also collected for this analysis. Annexin V Alexa Fluor 488 fluorescence was determined using a 488 nm blue laser and a 530/30 nm filter and PI fluorescence using a 488 nm violet laser and a 575/24 nm filter. On average, 10000 events are analyzed per sample. To correct the spectral overlap between Alexa Fluor 488 and PI, a process of fluorescence compensation was used, with the sample treated with staurosporine. This ensured to remove signal of each fluorochrome from the channel of the other one where it was also detected. A gating strategy was performed to define the target cell population to analyze: to exclude debris, but not dead cells, cells were gated on a two physical parameters dot plot measuring forward scatter (FSC) versus side scatter (SSC). The gated cell population was next analyzed in a dot plot with quadrant gates measuring Alexa Fluor 488 versus PI fluorescence.

### NGS

A fragment of mtDNA of 400-500bp surrounding the PstI restriction site located in the COX1 gene was amplified using Pfu DNA polymerase and primers containing Multiplex Identifiers (MIDs) for pooled sequencing. The sequences of the primers used are the following: WT-fwd 5’-CGT ATC GCC TCC CTC GCG CCA TCA GAC GAG TGC GTA GAC GTA GAC ACA CGA GCA T-3’; WT-rev 5’-CTA TGC GCC TTG CCA GCC CGC TCA GAC GCT CGA CAG CCG AGA AAG TGT TGT GGG A-3’; PstI-fwd 5’-CGT ATC GCC TCC CTC GCG CCA TCA GAG ACG CAC TCA GAC GTA GAC ACA CGA GCA T-3’; PstI-rev 5’-CTA TGC GCC TTG CCA GCC CGC TCA GAG CAC TCT AGG CCG AGA AAG TGT TGT GGG A-3’. PCR products were verified by electrophoresis on 1.2% agarose gels and purified using NucleoSpin Gel and PCR Clean-up according to the manufacturer’s protocol (Macherey-Nagel). The DNA concentration of each purified PCR product was determined using Qubit dsDNA HS Assay Kit (Molecular Probes). Equal amounts of PCR amplicons harboring different barcode sequences were pooled together in an equimolar ratio and subjected to pyrosequencing using a Roche 454 GS-FLX+ Titanium platform executed by Eurofins MWG (Ebersberg, Germany). Sequencing data were analyzed using the GS Data Analysis Software (Roche).

## Results

### A massive production of mtDNA DSBs triggers a rapid loss of mtDNA

**Fig 1:**
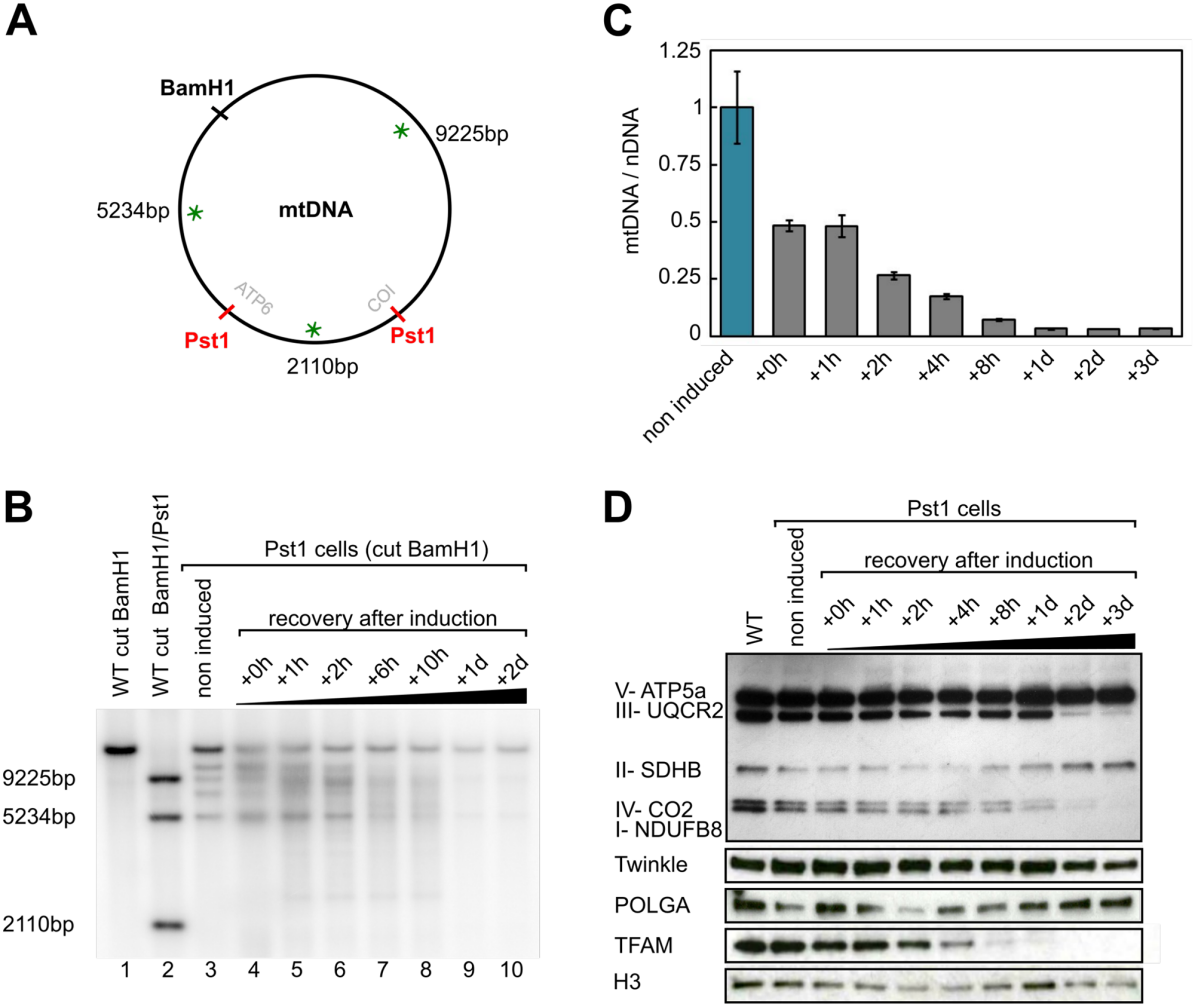
Loss of mtDNA after induction of the expression of a mitochondrial targeted PstI. **A**. Map of the human mtDNA indicating the positions of the unique BamHI site and the two PstI sites, the distances between these sites and the positions of the probes used for the Southern blots (green asterisks). **B**. Southern blot analysis of the control HEK293 cells (WT) and stably transfected cells (PstI cells) after digestion by BamHI or BamHI + PstI for the control. PstI expression was induced for 2h with doxycycline and the cells were followed during the recovery period, from 0h to 2 days after induction. **C**. Quantification of the mtDNA/nDNA ratios in the PstI stably transfected cells, without induction or from 0h to 3 days after a 2h induction with doxycycline (mean ± s.e., *N* = 3). **D**. Changes in mitochondrial proteins levels during the recovery period after induction of PstI for 2h with doxycycline. Total cell lysates (20 μg) were analyzed by western blot with antibodies against mitochondrial (CO2: complex IV) or nuclear encoded subunits of the respiratory chain (ATP5a: complex V; UQCR2: complex III, SDHB: complex II; NDUFB8: complex I) and against DNA maintenance proteins, the helicase Twinkle, the DNA polymerase POLGA and TFAM. H3: Histone H3B (loading control).

We were interested in understanding how mitochondria deal with the presence of DSBs in their genome. We thus needed a system to create DSBs specifically in the mitochondrial DNA. For this, we modified an existing system consisting of a restriction enzyme (PstI) fused to the mitochondrial targeting sequence of the human COX8 gene [36], and generated an inducible stable cell line (HEK293 FlpIn-TREx) expressing this enzyme. Using Southern blot, our first observation was that, even in the absence of induction with doxycycline, we always observed a basal leakiness of PstI expression (Fig 1B, lane 3). We then checked the efficiency of our system using 1 μg/mL of doxycycline for different induction times ranging from 5min to 10h (not shown). After induction, we observed (Fig 1B) the band corresponding to the full-length mtDNA (16569bp), and bands corresponding to the PstI total (9225 bp, 5234 bp and 2110bp), and partial (14459 bp and 7344 bp) digestions of mtDNA (Fig 1A), showing that our system is functional. We found that 2h was enough to obtain an almost complete digestion of the mtDNA, therefore we set this induction time for all the subsequent experiments.

To assess the effect of DSBs on mtDNA, we induced the expression of PstI for 2h and followed the cells for a recovery period of up to 3 days after induction. The expression of PstI results in the digestion of mtDNA, which is followed by an extremely rapid loss of mtDNA, as evidenced by the Southern blot (Fig 1B) and qPCR (Fig 1C) results. After 2h induction (+0h), there is already a decrease of ∼50% in the amount of mtDNA and, at 8h after induction (2h induction + 8h recovery), less than 10% of the mtDNA is left. Finally, between 1 and 3 days after induction, only a very small proportion of mtDNA (∼2%) still remains. This drastic reduction in the mtDNA content is accompanied by a loss of TFAM (-60% at t = 4h and -85% at t = 8h), which is not surprising as the amount of TFAM is strongly correlated to the amount of mtDNA present in cells [37, 38] (Fig 1D). Additionally, we observed a loss of the mitochondrial-encoded subunit CO2 and of the nuclear-encoded NDUFB8 and UQCR2 subunits of the respiratory chain (Fig 1D). This decrease takes place at a later stage (t = 2days), which might be due to a larger stability of these proteins. During the experiment, we also monitored the amount of the other respiratory chain subunits as well as the quantity of mtDNA metabolism proteins (the replicative helicase and polymerase, TWINKLE and POLG) and, surprisingly, we found no significant changes in the amount of these proteins during the time course of the experiment (Fig 1D).

### Depletion of 5 mitochondrial exo/endonucleases does not change the mtDNA-loss kinetics

**Fig 2:**
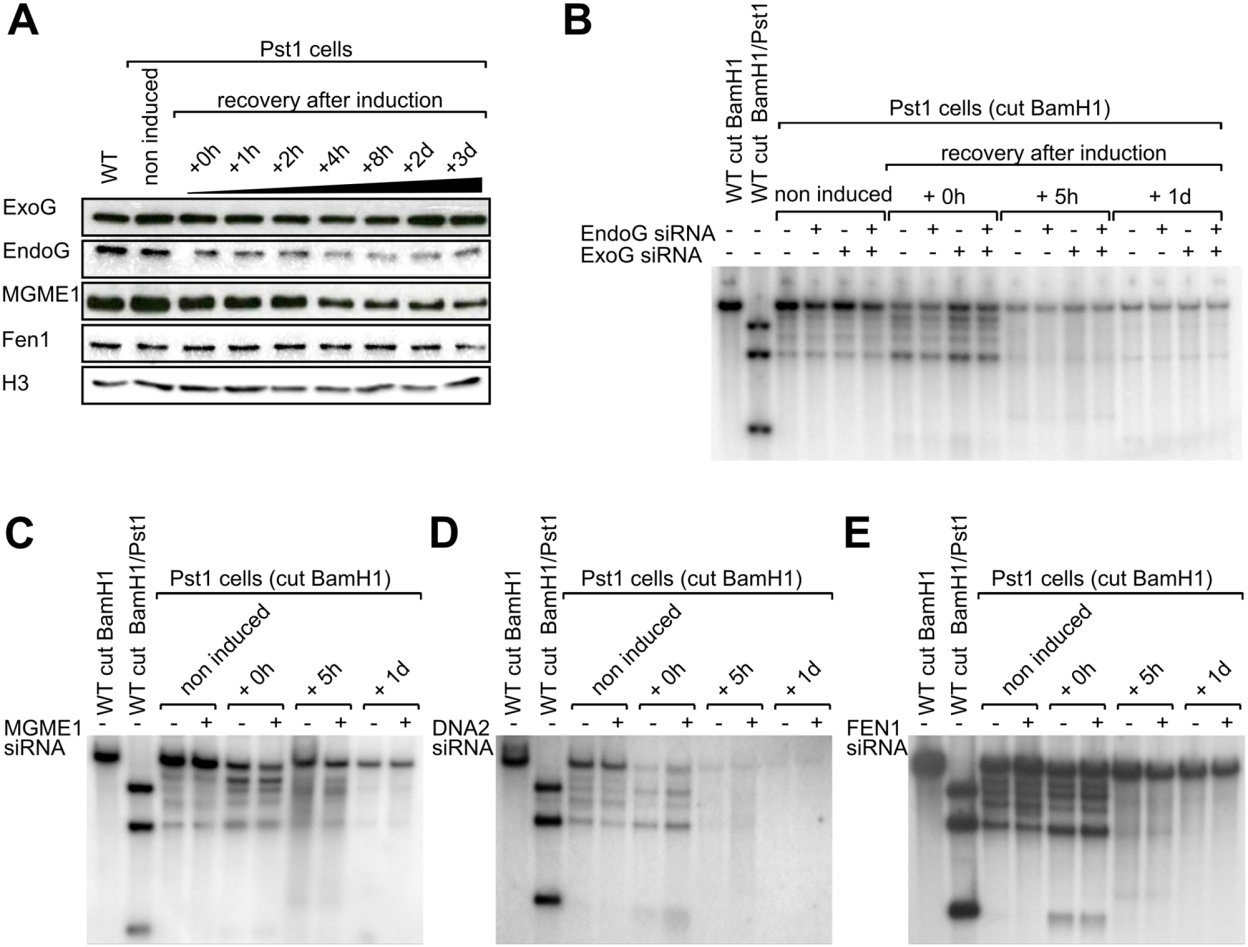
Effect of silencing different mitochondrial DNA nucleases on the rapid mtDNA depletion. **A**. Levels of different mitochondrial DNA nucleases before induction and during the recovery period after induction of PstI for 2h with doxycycline. Total cell lysates (20 μg) were analyzed by western blot with antibodies against ExoG, EndoG, MGME1, and FEN1. H3: Histone H3B (loading control). **B**. Southern blot analysis of the control HEK293 cells (WT) and stably transfected cells (PstI cells) after digestion by BamHI or BamHI + PstI for the control. The cells were grown in presence or absence of a mix of siRNAs targeting ExoG and/or EndoG. Three days after siRNA transfection, doxycycline was added to the cells for 2h to induce PstI expression, and the cell’s DNA was examined before induction and during the recovery period, at 0h, 5h and 1 day after induction. **C, D, E**. Similar experiments as in 2B, except that the siRNAs were targeting MGME1, DNA2 or FEN1, respectively.

Next, we evaluated the role of five known mitochondrial nucleases (EndoG, ExoG, MGME1, DNA2 and FEN1) in the fast mtDNA degradation process we observe after PstI digestion. We first followed by Western Blot the expression of these proteins for three days after induction of DSBs (Fig 2A). In the case of DNA2, the antibody gave inconclusive results, so for that nuclease we used RTqPCR (not shown). We did not observe an obvious increase in the amount of any of these proteins during the fast mtDNA degradation phase (between 0 and +6h after induction). On the contrary, it seems that the amount of EndoG and MGME1 slightly decreased in parallel to the mtDNA loss. Second, we used siRNA to individually knock down EndoG, ExoG, MGME1, DNA2 and FEN1. The efficiency of the siRNA was verified by Western Blot (or RTqPCR for DNA2) (S1 Fig). Three days after siRNA transfection, PstI expression was induced and the cell’s mtDNA was analyzed at time 0h, 5h and 1 day after induction (Fig 2B–2E). As observed previously, the expression of PstI results in a fast loss of mtDNA. However, we did not detect any modification of the kinetics of mtDNA loss upon siRNA knock-down of any of the targets. As we suspected a redundancy in the role of these proteins, we performed a double knock-down of ExoG and EndoG (Fig 2B) as well as a simultaneous knock-down of the five nucleases (S2 Fig). The results were however similar, i.e. no change in the kinetics of mtDNA loss. We thus conclude that these proteins are unlikely to be involved in the fast mtDNA loss that occurs after generating DSBs.

### mtDNA loss is not due to a loss of mitochondria

**Fig 3:**
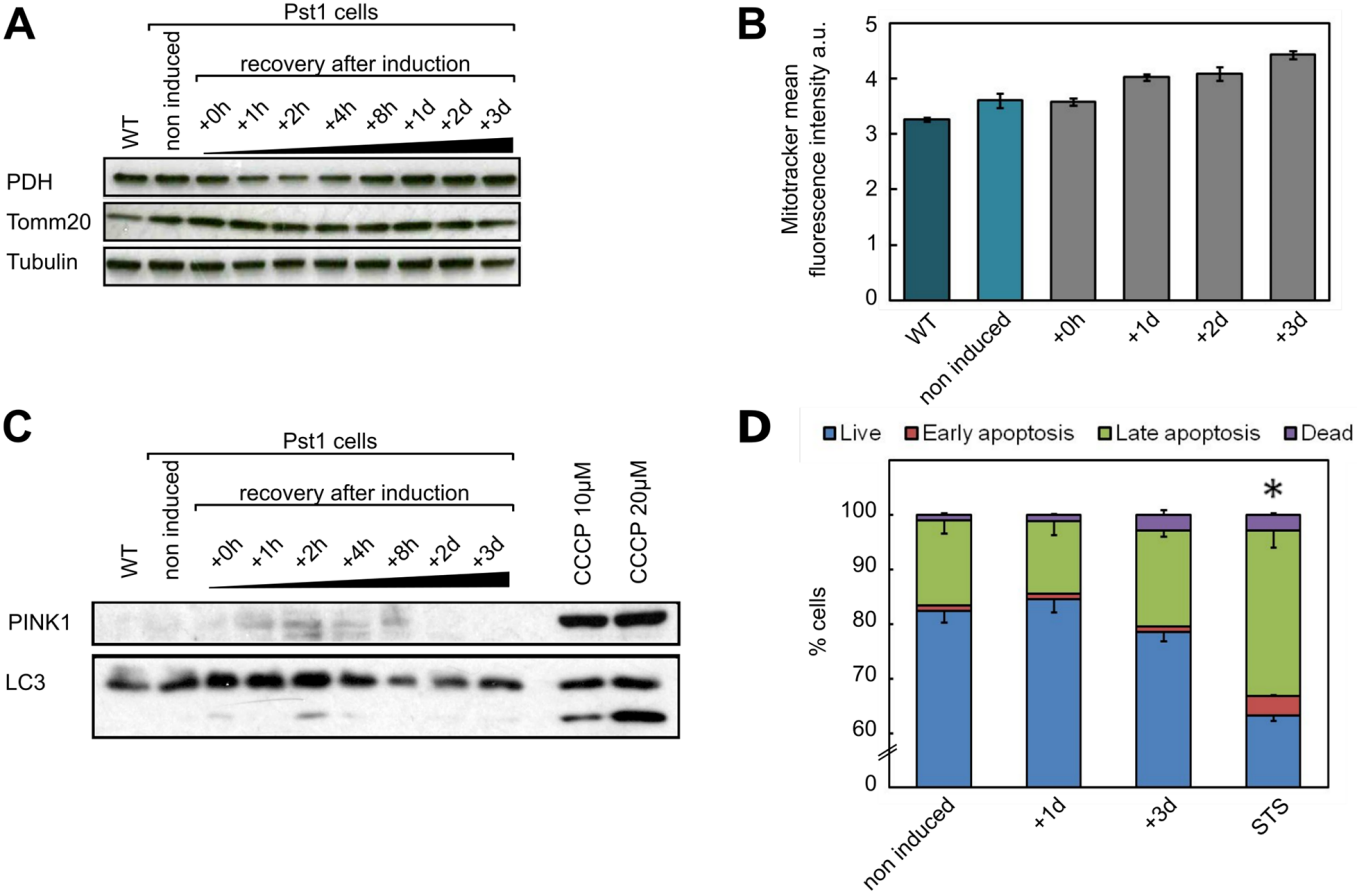
The rapid loss of mtDNA is not correlated to a loss of mitochondria, autophagy or apoptosis. Levels of **A**. mitochondrial proteins and **C**. an autophagy marker before induction and during the recovery period after induction of PstI for 2h with doxycycline. Total cell lysates (20 μg) were analyzed by western blot with antibodies against markers of the mitochondrial matrix (PDH), mitochondrial outer membrane (Tomm20), autophagy (LC3) and mitophagy (PINK1). CCCP is used as a positive control and Tubulin is used as loading control. Analysis of **B**. cell mitochondrial content and **D**. apoptosis by flow cytometry before induction and during the recovery period. * P ≤ 0.05 versus non-induced PstI cells for each cell population (Student’s t-test).

Currently, the general belief is that, when mtDNA is damaged, it is eliminated via autophagy/mitophagy [39, 40]. We thus set out to investigate if the loss of mtDNA we observed could be explained by a loss of mitochondria due to autophagy. First, using Western blot, we determined the amount of two mitochondrial markers, a mitochondrial matrix enzyme (PDH, pyruvate dehydrogenase) and a membrane protein (TOMM20, translocase of outer mitochondrial membrane). As shown on Fig 3A, the amount of these proteins remained unchanged up to 3 days after induction of DSBs, whereas mtDNA has almost completely disappeared after 8h after DSBs induction (Fig 1). Second, we estimated the mitochondrial mass by flow cytometry using MitoTracker Green. After induction of DSBs, we did not observe a decrease of the mitochondrial mass, instead, a slight increase could be observed at day 3 after DSBs induction (Fig 3B). To further elucidate if the mtDNA loss is due to autophagy/mitophgy, we estimated the amount of a marker of autophagy (LC3) and a marker of mitophagy (PINK1) by Western blot during the recovery period after DSBs induction (Fig 3C). As a positive control we used HEK293 WT cells treated for 18h with 10 μM or 20 μM CCCP. CCCP is an uncoupling agent known to induce generalized autophagy [41] and mitophagy [42]. Again, no change in the amount of these markers could be observed, supporting the hypothesis that that mtDNA degradation is not due to a degradation of mitochondria via autophagy/mitophagy. Finally, we also showed by flow cytometry using annexin V and PI staining that mtDNA degradation was not due to a loss of the cells by apoptosis (Fig 3D). These data show that mtDNA loss is not due to a loss of mitochondria by autophagy, mitophagy or apoptosis but may, on the contrary, trigger a slight increase of the amount of mitochondria, which could reflect a compensatory mechanism.

### Repopulation of cells with PstI-resistant mtDNA molecules

**Fig 4:**
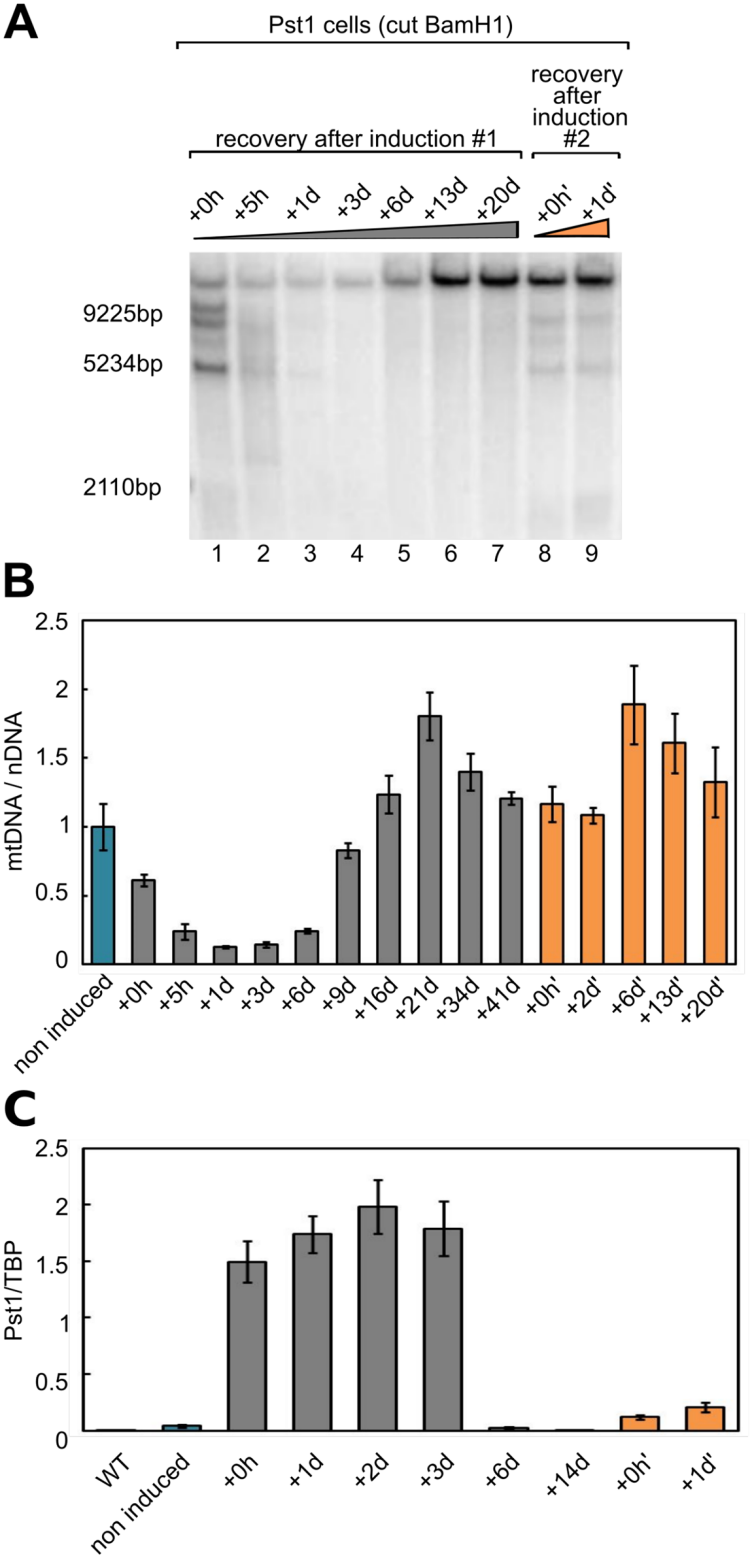
The loss of mtDNA is followed by a slow repopulation with intact mtDNA molecules. **A**. Southern blot analysis of the control HEK293 cells (WT) and stably transfected cells (PstI cells) after digestion by BamHI or BamHI + PstI for the control. PstI expression was induced for 2h with doxycycline and cells were followed for 20 days. At day 20, a second induction of 2h with doxycycline was performed and the cells were followed during the second recovery period (marked with an apostrophe). **B**. Quantification of the mtDNA/nDNA ratios in the PstI stably transfected cells, from 0h to 41 days after a 2h induction with doxycycline and from 0h to 20days after a second 2h doxycycline induction. (mean ± s.e., *N* = 3). **C**. Quantification of the level of PstI transcripts in the control HEK293 cells (WT) and stably transfected cells (PstI cells), during 2 sequential inductions of PstI expression with doxycycline (mean ± s.e., *N* = 3). TBP is used as a reference RNA.

Our results point to a rapid degradation of mtDNA as a consequence of a massive production of DSBs. However, although it remains debated, it has been suggested that human mitochondria have the capability of repairing DSBs via homologous recombination or NHEJ [12]. We thus decided to follow the cells for a longer period of time (up to 40 days after DSBs induction) to see if the degradation phase could be followed/accompanied by a repair mechanism. We observed that the initial period of mtDNA loss was followed by a slow return of full-length mtDNA molecules, starting around 6 days after DSBs induction (Fig 4A, lane 5). The amount of mtDNA slowly increased to a peak value roughly 20 days after induction (Fig 4B). Surprisingly, at this stage, we could observe only the full-length mtDNA molecules, and no partially or totally digested molecules. Moreover, a second induction with doxycycline at day 21 only produced a slight digestion of the mtDNA molecules (Fig 4B, orange bars). In order to find the cause of this apparent ‘resistance’ to PstI digestion, we first tested the presence of mutations in the PstI sites of the newly amplified mtDNA population. Mutations could be due to (i), a polymorphism in the PstI sites in the original mtDNA population that would have been selectively amplified, or (ii), a repair of some of the mtDNA molecules by an unfaithful repair mechanism (i.e. NHEJ) that would have created mutations within the PstI site. Using NGS, we sequenced a stretch of ∼100bp containing the PstI site located in the COX1 gene. We analyzed ∼40000 mtDNA molecules from PstI cells at day 13 after induction and compared them to ∼40000 from wild-type cells. We found that the only interesting mutation was an A to G transition that modifies the PstI site (Table 1). This mutation was more than 20 times more abundant in the PstI cells than in the wild-type cells (0.86% vs. 0.04%). However, although significantly increased compared to wild-type, this percentage of mutation within the PstI site cannot explain the apparent resistance to PstI observed by Southern blot, where a large majority of the mtDNA molecules (estimation 95%) is not digested by PstI. We next hypothesized that the apparent ‘resistance’ was caused by a loss of expression of PstI. We performed RTqPCR and found that, indeed, after 6 days, the expression of PstI has nearly vanished (Fig 4C). After a second induction (orange bars), a slight expression of PstI can be observed, close to the level of the leakage in the non-induced PstI cells, but much lower than the level of expression of PstI after the first induction (grey bars). We thus conclude that the newly amplified molecules are not ‘resistant’ to PstI digestion because of the amplification of a mutated version of the mtDNA (either due to polymorphism or repair) but to an extinction of PstI expression over time.

**Table 1:** Sequencing of the Pst1 site.

Pst1 sequence	C		T	G	C	A	G
*Mutation*	C/A	C/T	T/C	G/A	C/T	A/G	G/A
*WTcells*	0%	0.04%	0.02%	0.06%	0.02%	0.04%	0.05%
*Pst*1*cells*	0.01%	0%	0%	0.02%	0.04%	0.86%	0.01%

Sequencing was performed for the control cells (WT) and stably transfected cells (Pst1) 13 days after Pst1 induction (∼40 000 reads for each).

## Discussion

In this study, we investigated the consequences of the production of mitochondrial DSBs. *In vivo*, these damages can be generated by replication fork arrest, endogenous or exogenous sources such as exposure to ROS produced during the oxidative phosphorylation, viruses, ultraviolet and ionizing radiation or other clastogens. To create mtDSBs in a controlled way, we used an inducible system targeted to the mitochondria. Cells were stably transfected with the restriction enzyme PstI fused to a mitochondrial targeting sequence and under the control of a promoter inducible with doxycycline. After doxycycline induction we efficiently obtained DSBs specifically on mtDNA, located at the PstI restriction sites. The use of an inducible system has the advantage to control the amount of mtDSBs created. However, the cells that we used showed a background expression of PstI even in the absence of the inducer. This basal leakiness was present in all the clonal populations we checked even thought we used tetracycline-free serum. Transgene leakage in inducible overexpression systems is a recurrent problem [43]. In our case, even if the leakiness enabled us to study the impact of a low amount of mtDSBs on the cells and on mtDNA (which would not be possible with a constitutive system), it questions the efficiency of such an inducible system in conditions where a total on/off system is required. Nonetheless, as even the lowest possible PstI expression can be expected to give at least some mtDNA digestion, the analysis of non-induced cells show this has very little effect on cell growth, even when the cells were cultivated in low glucose or galactose media, showing that cells can thus cope well with this level of damage. It is also interesting to note that in our system, a second induction with doxycycline following the first digestion and a period of recovery, led to almost complete loss of expression of PstI. The reasons of this silencing are still unclear, but it could be that epigenetics mechanisms come into play in response to the damage caused to the cells after the first induction in order to inactivate the foreign gene. It is also possible that some cells underwent rearrangement in the PstI coding sequence/regulatory regions and thus lost their inducibility.

Data on mammalian mtDNA repair after DSBs are rather controversial. In mouse, there are suggestions of mtDNA deletions caused by mtDNA recombination events following DSBs induction by the mitochondrial-targeted endonucleases ScaI [13] and PstI [36]. In both cases, a region close to the D-loop was involved in most recombination events. On the contrary, in human osteosarcoma cells, no recombination could be detected at significant level after the use of PstI targeted to mitochondria [31]. *In vitro* studies on digested plasmids have also indicated the ability of human and rodent mitochondria to perform MMEJ, the efficiency of the recombination being increased with the length of homology [14, 29]. On the contrary, it has also been suggested that the formation of deletions was not due to MMEJ or homologous recombination but was the result of a repair mechanism coupled to mtDNA replication and mediated by the replisome [44]. In our experiments, the induction of PstI expression resulted in the generation of a massive amount of DSBs. The majority of the cut mtDNA molecules was rapidly degraded but could, after a recovery time, slowly repopulate the cells. In order to understand this repopulation, we sequenced 100 bp of mtDNA containing one of the two PstI sites. Interestingly, we did not find any mutations or short deletions that could suggest repair by NHEJ or MMEJ. The only difference we could notice after DSBs generation was an increase in the proportion of a point mutation in the PstI restriction site, probably reflecting an amplification of a pre-existing polymorphism. Finally, after induction of mtDSBs we observed an almost total loss of mtDNA, which argues against an efficient repair system of DSBs in our cell model (HEK293) upon generation of a massive amount of DSBs. Although it is impossible to completely rule out that homologous recombination (which is error-free) did not occur in our system, it seems very unlikely or must be a very rare event since most of the mtDNA molecules were rapidly degraded.

After generation of a large amount of mtDSBs we observed a massive and rapid loss of the mitochondrial genome (Fig 1B & 1C). Our data are in line with previous studies using restriction endonucleases targeted to mitochondria, showing either a shift from heteroplasmy to a unique mtDNA haplotype, not recognized by the restriction enzyme [45, 46] or a total or almost total loss of the mitochondrial genome, in case of homoplasmy [31, 32]. A total loss of mtDNA (generation of *ρ*^0^ cells) was however only obtained in the case of a constitutive expression of the enzyme [32]. An important depletion of mtDNA followed by a repopulation after withdrawal of the inducer was also observed when damages to mtDNA were generated with the use of the bacterial exonuclease III, which creates single-strand breaks [17]. One hypothesis that can explain the loss of mtDNA is the elimination of the damaged mitochondria via autophagy/mitophagy or by massive apoptosis. Several studies point in this direction in different organisms. For instance, in the nematode, knockdown studies showed that removal of helix-distorting mtDNA damage caused by UVC exposure involved genes in autophagy and mitochondrial dynamics [47]. In mammalian cells, mitophagy triggered by an overexpression of Parkin has been shown to be responsible for the elimination of mitochondria containing mtDNA with deleterious mutations [48]. Similarly, upregulation of mitochondrial dynamics and mitophagy has been shown to correlate with efficient removal of a pathological mutant mtDNA in a cell-type-dependent manner [49]. However, the rate of mtDNA loss we observed here is extremely fast (starting within 2h, almost complete after 1 day), while we only observed slight alterations in apoptosis and mitochondrial mass at the later stages during recovery after induction (Fig 3B & 3D). Quantification of LC3 and PINK1 during the kinetics of mtDNA loss also showed no increase of these markers of autophagy and mitophagy (Fig 3C), confirming that these mechanisms are unlikely responsible for the loss of mtDNA in our system. The massive and rapid loss of mtDNA is therefore not due to elimination of the whole mitochondria but probably due to a still unknown mechanism directly processing damaged mtDNA. The rate of mtDNA degradation we observed is consistent with results published by Shokolenko *et al*., who detected a substantial elimination of mitochondrial genome after 6h to 1day after induction of mutated UNG1 or exoIII expression (generation of abasic site or single-strand breaks) [17]. This fast rate of mtDNA loss cannot be due to a blockage of mtDNA replication, as mtDNA loss following the expression of a dominant inactive form of the replication DNA polymerase (POLG) takes more than 6 days in HEK cells [50].

To test the hypothesis of more direct (and enzymatic) degradation of mtDNA following DSBs in our system, we searched for proteins that could be involved in this process and therefore we tested the five DNA nucleases known to be present in the mitochondria. Our two first obvious candidates were ExoG and EndoG. ExoG is solely located in the mitochondria and is the major 5’-exonuclease for mitochondrial LP-BER and single-strand break repair. Cells depleted in ExoG accumulate more DNA damages and develop mitochondrial dysfunctions triggering apoptosis [20]. EndoG has been well studied over the last decades, but its molecular function in mitochondria is still unclear. It plays a role in DNA metabolism in both mitochondria and nucleus, in various processes such as apoptosis [51] or nuclear recombination [52]. Recently its crystal structure in complex with DNA demonstrated that it has a strong non-specific endonuclease activity [53]. It has also been suggested that these two proteins could contribute to the mtDNA degradation observed following HSV infection [54]. FEN1 and DNA2 are believed to be involved in BER pathway in both nucleus and mitochondria. They can process intermediate 5’ flap structures during DNA replication and long-patch BER [26, 27]. MGME1 is also implied in primer processing occurring in mtDNA replication [23]. Its depletion leads to accumulation of mitochondrial 7S and intermediates of stalled replication [55]. Here, using a knockdown approach, the depletion of these enzymes did not change the kinetics of mtDNA loss, showing that these proteins are unlikely to be involved in the removal of linear mtDNA fragments created after mtDSBs.

## Conclusion

The redundancy of the mitochondrial genome can explain why cells are quite tolerant to mtDNA depletion and can lose a substantial proportion of their mtDNA without impeding mitochondrial function. We observed that the cells used in this study can survive a major deficiency in mtDNA, even when we cultivate them in low glucose or in galactose media (not shown). Our work argues against the existence of a DSB repair pathway in mitochondria and instead supports the existence of a pathway for the targeted degradation of damaged mtDNA in a mitophagy/autophagy-independent manner. Future studies will reveal the identity of the proteins involved.

## Supporting information

S1 Fig Western blot of the silenced proteins. Cell pellets were collected for Western blot 72h post-transfection with siRNA against **A**. EndoG, ExoG, **B**. MGME1 and **C**. FEN1.
(TIF)

S2 Fig Effect of the simultaneous silencing of five mitochondrial nucleases on the rapid mtDNA loss. Southern blot analysis of the control HEK293 cells (WT) and stably transfected cells (PstI cells) after digestion by BamHI or BamHI + PstI for the control. The cells were grown in presence or absence of a mix of siRNAs targeting ExoG, EndoG, FEN1, DNA2 and MGME1. Three days after siRNA transfection, doxycycline was added to the cells for 2h to induce PstI expression, and the cell’s DNA was examined before induction and during the recovery period, at 0h, 5h and 1 day after induction.
(TIF)
