# Supplementary figures and images for: Selective mitochondrial DNA degradation following double-strand breaks
Source: PLoS One. 2017 Apr 28;12(4):e0176795. doi: 10.1371/journal.pone.0176795 (PMC5409072; doi:10.1371/journal.pone.0176795)

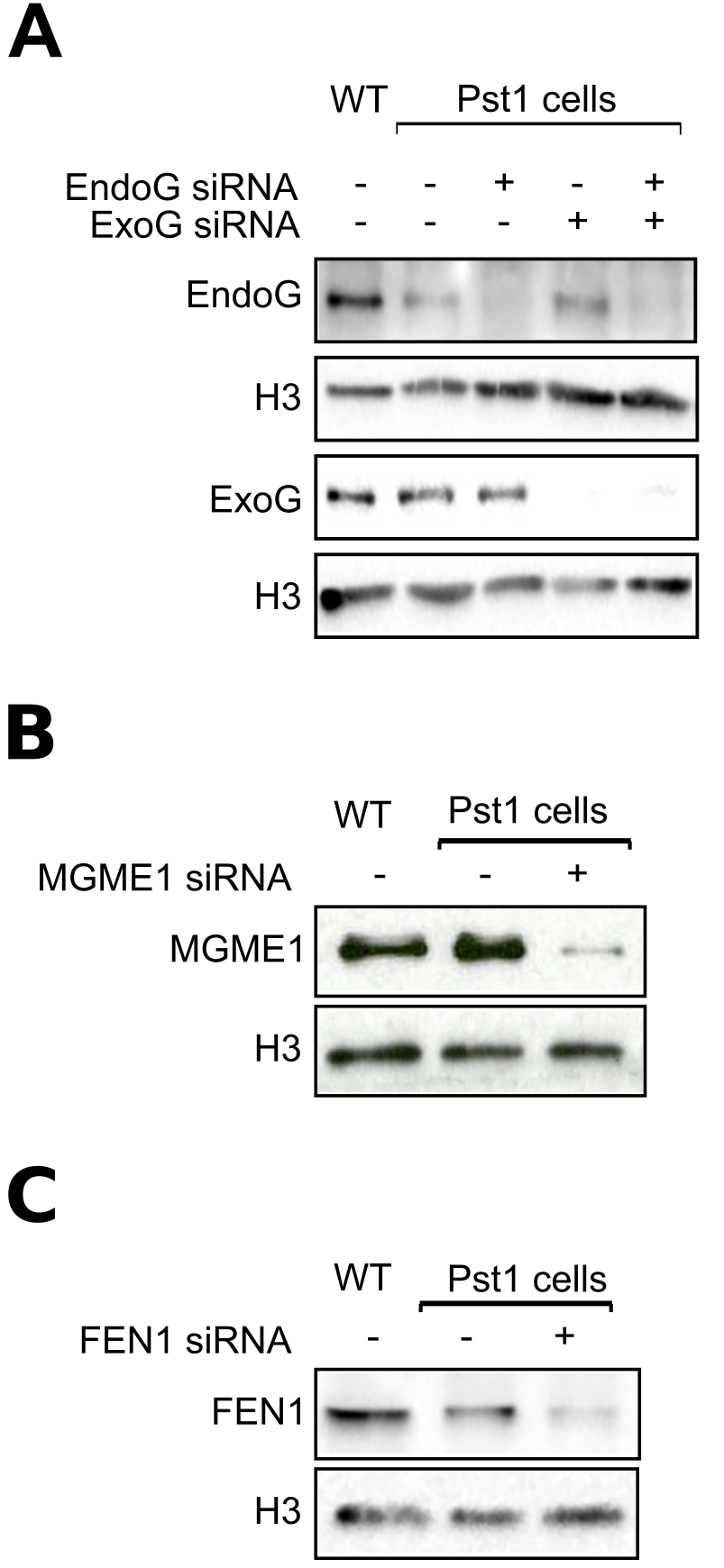

Supplement: S1 Fig — Cell pellets were collected for Western blot 72h post-transfection with siRNA against A. EndoG, ExoG, B. MGME1 and C. FEN1. (TIF) [file pone.0176795.s001.tif]

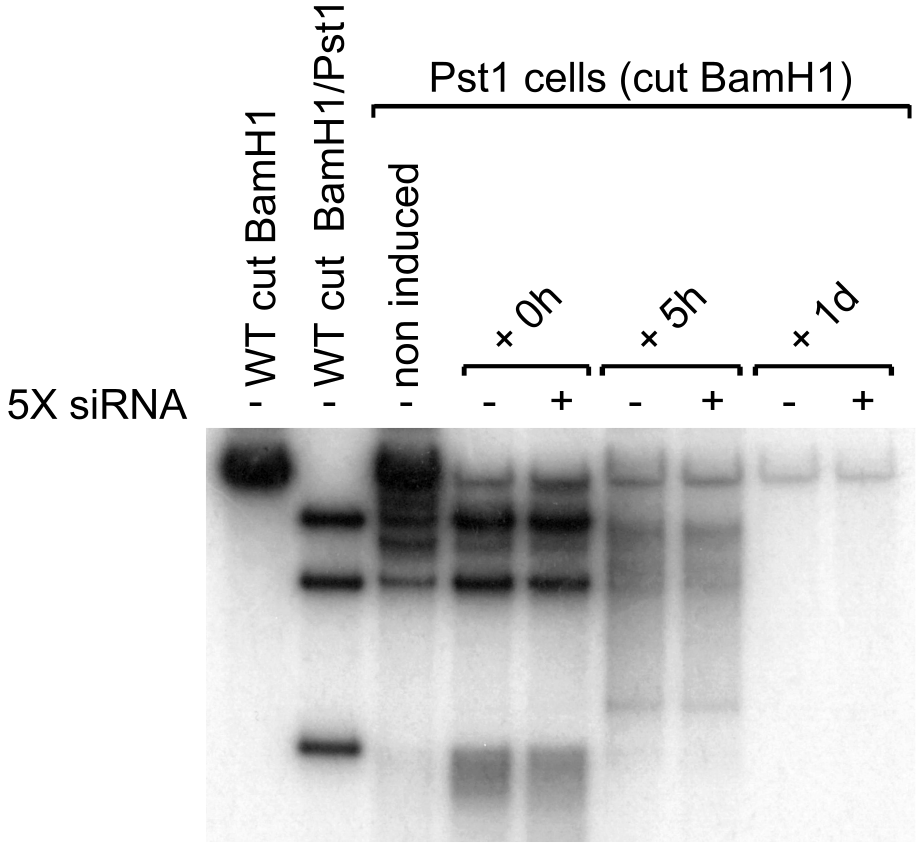

Supplement: S2 Fig — Southern blot analysis of the control HEK293 cells (WT) and stably transfected cells (PstI cells) after digestion by BamHI or BamHI + PstI for the control. The cells were grown in presence or absence of a mix of siRNAs targeting ExoG, EndoG, FEN1, DNA2 and MGME1. Three days after siRNA transfection, doxycycline was added to the cells for 2h to induce PstI expression, and the cell’s DNA was examined before induction and during the recovery period, at 0h, 5h and 1 day after induction. (TIF) [file pone.0176795.s002.tif]
